# Circ-ZEB1.33 promotes the proliferation of human HCC by sponging miR-200a-3p and upregulating CDK6

**DOI:** 10.1186/s12935-018-0602-3

**Published:** 2018-08-13

**Authors:** Yuhua Gong, Jinzhong Mao, Di Wu, Xuemei Wang, Long Li, Liang Zhu, Rong Song

**Affiliations:** 1 Department of Clinical Laboratory, The Third People’s Hospital of Zhenjiang, 300 Daijiamen, Zhenjiang, 212005 Jiangsu China; 2 Department of Radiology, The Third People’s Hospital of Zhenjiang, 300 Daijiamen, Zhenjiang, 212005 Jiangsu China; 3 Department of Hepatosis Inpatient, The Third People’s Hospital of Zhenjiang, 300 Daijiamen, Zhenjiang, 212005 Jiangsu China

**Keywords:** Circ-ZEB1.33, miR-200a-3p, CDK6, HCC

## Abstract

**Background:**

Accumulating data indicated that circRNA plays important roles in regulating many biological processes of the tumor, the present study is designated for exploring roles of the circ-ZEB1.33-miR-200a-3p-CDK6 regulating axis in human hepatocellular carcinoma (HCC).

**Methods:**

The regulation axis as predicted by using online tool circNet, the expression and correlation of circ-ZEB1.33-miR-200a-3p-CDK6 was verified in human HCC. The diagnostic value of both tumor and serum circ-ZEB1.33 was estimated by using clinical samples. The roles of circ-ZEB1.33-miR-200a-3p-CDK6 in regulating cell cycle were explored by using in vitro studies.

**Results:**

Overexpression of circ-ZEB1.33 and CDK6, downregulation of miR-200a-3p were detected in human HCC tissues, negative correlation between circ-ZEB1.33 and miR-200a-3p, positive correlation between circ-ZEB1.33 and CDK6 were confirmed in human HCC tissues. Tissue and serum circ-ZEB1.33 were related to different TMN stages and prognosis in HCC patients. RNA pull-down assay implied that circ-ZEB1.33 could decrease miR-200a-3p by sponging miR-200a-3p, and the luciferase reporter assay indicated that miR-200a-3p could downregulate CDK6 transcription by targeting its 3′UTR. The in vitro assays indicated that circ-ZEB1.33 could promote the proliferation of HCC cells by increasing the percentage of S phase regulated by CDK6/Rb.

**Conclusion:**

Proliferation promotion roles of the circ-ZEB1.33-miR-200a-3p-CDK6 regulating axis are existed and verified in human HCC, both tumor and serum circ-ZEB1.33 can serve as an indicator for the prognosis of HCC patients.

**Electronic supplementary material:**

The online version of this article (10.1186/s12935-018-0602-3) contains supplementary material, which is available to authorized users.

## Background

Circular RNA (circRNA) is a type of RNA capable of forming a covalently closed continuous loop [1, 2]. CircRNAs are mostly non-translated, can occur in any genomic region including gene-bearing regions and intergenic regions, and range in length from few hundred to thousands of nucleotides [3]. CircRNAs are resistant to exonuclease-mediated degradation due to their characters in the absence of 5′ or 3′ ends. Therefore, they are presumably more stable. Regarding the function of circRNAs, it can be briefly categorized into several aspects, including downregulating miRNA by absorbing or sponging them, regulation their parental genes transcription, serve as a biomarker for age, cancer, etc. Among all of the functions listed above, miRNA sponging is the one of the functions discovered by Memczak et al. at the first time, revealing that circRNA could act as a sponge to bind and sequester miRNA in a sequence-specific manner [2]. Since the well-accepted function of miRNA in cancer, many attentions have been drawn concerning the roles of circRNAs in initiation and progression of cancer [1–3]. Decoding circRNAs interplay with other RNA species in cancer would likely confer circRNAs great potential to become new diagnostic markers in cancer diagnosis and novel therapeutic interventions [3].

Since the better stability, serum circRNA can be detected in the peripheral serum, and make them ideal biomarkers for disease diagnosis or prediction [1]. Increasing amount of related research has been published addressing the diagnosis value of circRNA in the diseases including hypertension, lupus nephritis, diabetic retinopathy as well as liver cancer.

Expression patterns in CircNet, an online circular RNA regulatory prediction tool, indicated that four identified circRNAs: circ-ZEB1.5, circ-ZEB1.19, circZEB-1.17, and circ-ZEB1.33 were down-regulated in human lung cancer specimens compared to the normal lung tissue samples, and all four circRNAs have the potential to sponge the miR-200 [4]. However, in our preliminary data, we found that circ-ZEB1.33 was overexpressed not only in human HCC tissues, but also patients’ serum. Moreover, circ-ZEB1.33 was well negatively correlated to the miR-200a-3p in the tumor. Therefore, in this study, we sought to investigate the diagnostic value of circ-ZEB1.33 as well as its mechanism regulating the cell proliferation of HCC cells through sponging miR-200a-3p.

## Materials and methods

### Patients

The hospital-based case–control study consists of 64 HCC patients and 30 cancer-free controls. All the subjects were recruited from the third People’s Hospital of Zhenjiang between January 2012 and September 2016. All patients were enduring surgery treatment for primary HCC, anyone with other hematological disorders, previous history of cancers, and chemotherapy were excluded. The cancer-free control subjects from the same geographic area showed no evidence of a genetic relationship with the cases. This study was approved by the Ethics Review Board of the Third People’s Hospital of Zhenjiang, and every patient had written informed consent. The clinical features of all the cases and controls were presented in Table 1.

**Table 1:** Clinical characteristic of HCC patients and cancer-free controls

Features	Cases (*n* = 64)		Controls (*n* = 30)		P*	*P* ^*#*^
	*N*	%	*N*	%		
Age (years)					0.805	0.743
≤ 50	36	56.25	16	53.33		
> 50	28	43.75	14	46.67		
Gender					0.248	0.321
Male	42	65.63	15	50.00		
Female	22	34.38	15	50.00		
HBV infection					< 0.0001	0.145
Positive	58	90.63	5	16.67		
Negative	6	9.38	25	83.33		
TMN stage						0.012
I	12	18.75				
II	22	34.38				
III	17	26.56				
IV	13	20.31				
Tumor size (cm)						0.004
≤ 5	37	57.81				
> 5	27	42.19				
Metastasis						0.143
Yes	27	42.19				
No	37	57.81				

* Two-sided Chi square test for different features between cases and controls

^#^ The student’s t-test for circ-ZEB1.33 expression among different features of HCC patients

### Potential circRNA–miRNA-gene regulatory prediction

The possible interaction between circ-ZEB1.33 and sponge miRNA was predicted by using online prediction tool, CircNet (http://circnet.mbc.nctu.edu.tw/).

### Cell lines and reagents

Human HCC cell lines including 97H, Huh7, HepG2, SNU423, SNU475, and L02 were purchased from American Type Culture Collection (ATCC). All cells were cultured in Dulbecco modified Eagle medium (DMEM) purchased from Gibco (CA, USA) supplemented with 10% fetal bovine serum (Invitrogen, Carlsbad, USA) and maintained in humidified 5% CO_2_ at 37 °C.

### Real-time PCR

The total serum RNA was extracted from 3 ml HCC and normal control serum by using GenEluteTM Plasma/Serum RNA Purification Mini Kit (Cat. RNB500) (Sigma-Aldrich, MO). For the HCC and adjacent tissues, the total RNA was isolated with TRIzol reagent. And the expression of circ-ZEB1.33 was detected by using Sybergreen^®^ based real-time PCR. The primers for genes involved in the study were: forward primer: CAGACTCTCCTGAGAAGAA, reverse primer: AAAGCCTCACTGAAAGGAAACA, amplicon size 127 bp for circ-ZEB1.33, and forward Primer TGTGGGCATCAATGGATTTGG, reverse Primer ACACCATGTATTCCGGGTCAAT, amplicon size 110 bp for GAPDH. The transcription of miR-200a-3p was detected by using a commercial Taqman probe (Assay ID: Hs04231538_s1) (Thermofisher). The miR-200a-3p inhibitor was also purchased from Thermo Fischer (MH10991).

### Western blot

Cells were collected and lysed with cell lysis buffer (RIPA) for western blotting (Beyotime, Haimen, China) containing 150 mM NaCl, 1.0% IGEPAL^®^ CA-630, 0.5% sodium deoxycholate, 0.1% SDS, 50 mM Tris, pH 8.0. The proteins (30 μg per lane) were separated on 12% SDS-polyacrylamide gels and transferred on a polyvinylidene fluoride membrane (Millipore, Billerica, MA, USA). Immunoblotting of the membrane was performed using the following primary antibodies: anti-CDK6 (ab124821), pRb (s795) (ab47474), Rb (ab24) β-actin (ab8227), all the antibodies were purchased from Abcam (Cambridge MA).

### RNA pull-down

The RNA pull-down assay was performed with Pierce Magnetic RNA–Protein Pull-Down Kit (Thermofisher, CA) in accordance with the instructions from the manufacturer. Briefly, the total RNA was extracted from 97H and Huh7 cell, after the treatment of RNase in room temperature, magnetic beads were incubated with Probes for biotin-labeled circ-ZEB1.33 (Genescript Co., Nanjing, China) and U6 control. The relative expression levels of miR-200a-3p, circ-ZEB1.33 and U6 in the extract were detected by RT-PCR.

### Immunohistochemistry staining

Tissue sections were deparaffinized and rehydrated with a graded ethanol series and distilled water, and then treated with 3% H_2_O_2_ in methanol for 30 min to block endogenous peroxidase activity. Tissue sections were then rinsed twice for 5 min in phosphate-buffered saline (PBS) and incubated with 10% normal goat serum for 30 min to block non-specific antibody binding. After washing, the samples were incubated with a primary anti-CDK6 (ab124821, Abcam, MA) at 4 °C overnight. Sections were then washed in PBS three times and incubated with secondary antibodies. The sections were then stained with DAB per the manufacturer’s protocol, mounted on slides, and photographed using a digital microscope camera (Nikon, Tokyo, Japan).

### Cell cycle analysis

The cells were washed in phosphate-buffered saline (PBS) and fixed in 75% ice-cold ethanol at − 20  °C overnight. After rehydration with ice-cold PBS, cells were stained with PI/RNase Staining Buffer (BD Biosciences, San Jose, CA, USA) and analyzed using flow cytometry on a FACSCalibur Flow Cytometer (BD Biosciences) using the CellQuest Pro software (BD Biosciences).

### Cell proliferation assay

The proliferation assay was performed in a Plate 96 by planting initial cell number of 2 × 10^3^ and detected by using a CCK8 (Dojindo Molecular Technologies, Inc, Japan) kit according to the manufacturer’s protocol.

### Luciferase reporter assay and transfection of miR-200a-3p

The 3′UTR region of CDK6 containing the wild-type or mutant potential target site for miR-200a-3p was synthesized in Genescript Co. (Nanjing, China) and inserted into the pGL4.10[luc2] Vector (Promega, WI). For luciferase assay, Huh7 were co-transfected pGL4-CDK6-WT 3′UTR or pGL4-CDK6-MU 3′UTR, with miR-200a-3p mimics or control (GenePharma, China) using Lipofectamine 2000 (Thermofisher, CA). Cells were harvested 48 h after transfection for luciferase assay using a Dual-Luciferase^®^ Reporter Assay System (Promega, WI) according to the manufacturer’s protocol.

### Statistical analysis

Data are presented as mean ± SD. χ^2^ tests and the Student’s t-test analysis of variances were used to evaluate statistical differences in demographic and clinical characteristics. The two values correlations were evaluated by Linear correlation analysis and tested F test. The overall survival in different groups was analyzed by using Kaplan–Meier curve. All the expression experiments we conducted in vitro were repeated at least three times with samples in triplicates. Statistical analysis was performed using the GraphPad Prism software (CA, USA). In all cases, P < 0.05 was considered significant.

## Results

### Overexpression of Circ-ZEB1.33 in human tumor tissues and serum in HCC patients

**Fig. 1:**
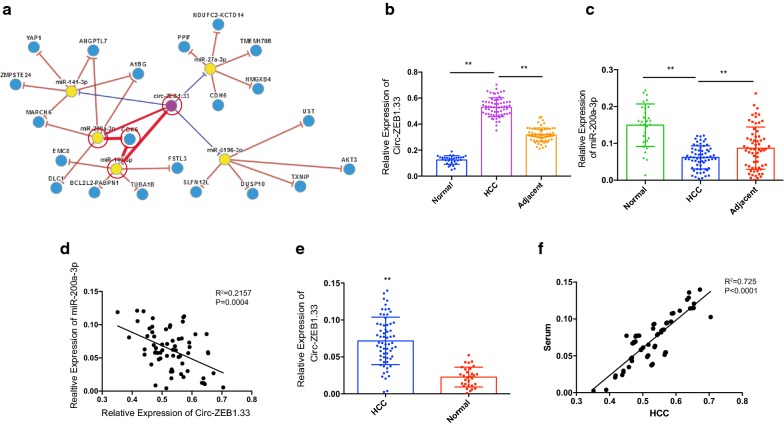
Overexpression of Circ-ZEB1.33 in human tumor tissues and serum in HCC patients. **a** CircNet prediction of circ-ZEB1.33 regulation of miR-200a-3p and miR-140-3p targeting CDK6. The predicted regulatory links among cicRNA (purple dots), miRNA (yellow dots) and genes (blue dots) was listed, among which the regulatory axis of cir-ZEB1.33-miR-200a-3p/miR-1403p-CDK6 was emphasized using bold red line. **b**, **c** The transcription of circ-ZEB1.33 and miR-200a-3p in paired HCC tumor, adjacent tissues (n = 64) and normal liver tissues (n = 30) by real-time PCR. The relative-expression of circ-ZEB1.33 and miR-200a-3p were calculated using 2−ΔΔct methods compared to GAPDH and U6 respectively. **d** Linear correlation between miR-200a-3p and circ-ZEB1.33 in human HCC tumor tissues. **e** The existence of circ-ZEB1.33 in HCC and health control serum was detected by using real-time PCR. **f** Linear correlation between tumor and serum circ-ZEB1.33 in human HCC patients. The data were presented as mean ± SD, *P < 0.05, **P < 0.01, by Student’s t-test

We queried the possible interaction between circ-ZEB1.33 and sponge miRNA by using online prediction tool, CircNet (http://circnet.mbc.nctu.edu.tw/), we found that circ-ZEB1.33 might promote CDK6 expression by sponge miR-140-3p and miR-200a-3p (Fig. 1a). We first verified this circRNA–miRNA-gene regulatory network in human hepatocellular carcinoma by using 64 paired HCC-adjacent tissues and normal control. We found that circ-ZEB1.33 overexpressed in tumor tissues compared to the adjacent tissues as well as normal liver tissues (Fig. 1b). We also found that it is miR-200a-3p but not miR-140-3p significantly decreased in tumor tissues compared to the adjacent tissues as well as the normal tissues (Fig. 1c). Furthermore, we performed a correlation study between the expression of circ-ZEB1.33 and miR-200a-3p in tumor tissues, significantly negative correlation was found (Fig. 1d). Furthermore, we also detected the existence of circ-ZEB1.33 in the serum of HCC patients and healthy control. The results indicated that the serum level of circ-ZEB1.33 was also significantly higher in HCC patients compared to the healthy control (Fig. 1e). A good correlation was found between the circ-ZEB1.33 value of serum and tumor tissue (Fig. 1f).

### Tissue and serum circ-ZEB1.33 can serve as a biomarker in diagnosis and prediction the prognosis of HCC

**Fig. 2:**
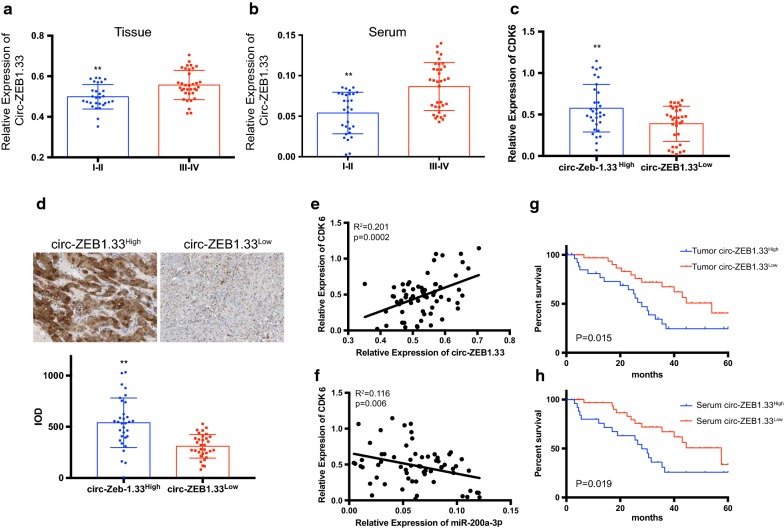
Tissue and serum circ-ZEB1.33 can serve as a biomarker in diagnosis and prediction the prognosis of HCC. The comparison between the expression level of circ-ZEB1.33 in tumor tissues and patients’ serum were presented in **a** and **b** respectively. **c**, **d** The transcription and protein expression of CDK6 in tumor tissue circ-ZEB1.33 High and low subgroup were determined by real-time PCR and IHC respectively. For integrated optical intensity (IOD) value estimation, the random five figures of each slide were analyzed by using image J. **e**, **f** the linear correlation between the transcription of CDK6 and circ-ZEB1.33 or miR-200a-3p in HCC tissues were analyzed. **g**, **h** the survival comparisons according to the expression of circ-ZEB1.33 in the tumor (**g**) and serum (**h**) were performed by using Kaplan–Meier curve. The data were presented as mean ± SD, *P < 0.05, **P < 0.01, by Student’s t-test

The circ-ZEB1.33 transcription in both tumor tissues and serum also compared based on different TMN stage, the results indicated that circ-ZEB1.33 was higher in the III–IV stage compared to the I–II stage (Fig. 2a and b). We further divided the HCC patients into two groups according to the expression of circ-ZEB1.33 in tumor tissues. The expression of miR-200a-3p, as well as its potentially targeting gene CDK6, were detected. Supporting the results obtained from Fig. 1, the transcription of miR-200a-3p significantly lower in circ-ZEB1.33^High^ group compared to circ-ZEB1.33^Low^ group (Fig. 2c). The transcription and protein expression of CDK6 also significantly higher in the circ-ZEB1.33^High^ group compared to circ-ZEB1.33^Low^ group (Fig. 2d and e). A negative correlation between miR-200a-3p and CDK6 transcription and a positive correlation between circ-ZEB1.33 and CDK6 transcription were found within HCC tumor tissues (Fig. 2e and f). Also, the 5-year survival rate was compared between the circ-ZEB1.33^High^ and the circ-ZEB1.33^Low^ groups based on tissue and serum circ-ZEB1.33 level respectively, the significantly lower survival rate was found in the circ-ZEB1.33^High^ compared to the circ-ZEB1.33^Low^ group (Fig. 2g and h).

### Circ-ZEB1.33 can sponge miR-200a-3p and attenuate the downregulation of CDK6

**Fig. 3:**
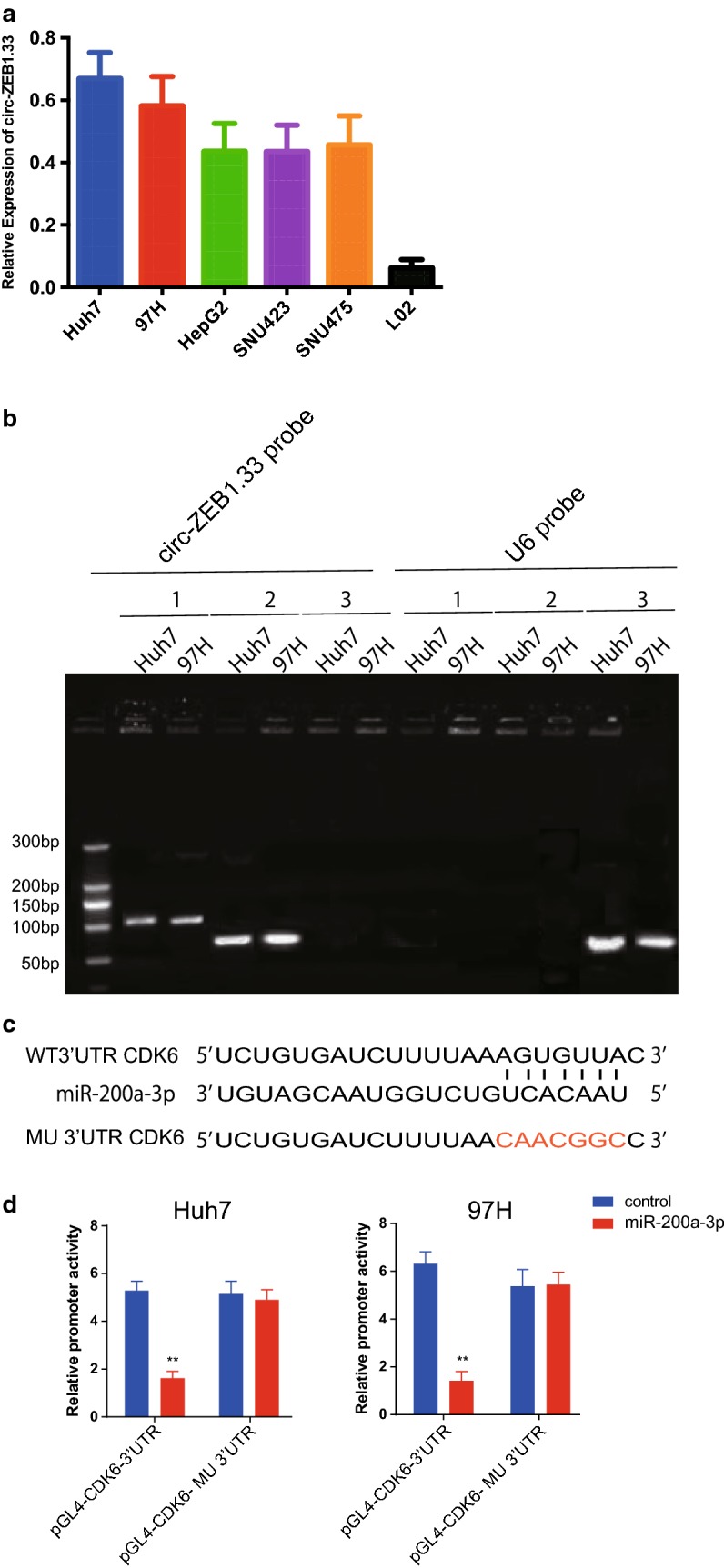
Circ-ZEB1.33 can sponge miR-200a-3p and attenuate the downregulation of CDK6. **a** The expression screen of circ-ZEB1.33 in different human HCC cell lines were performed by using real-time PCR. **b** A RNA pull-down assay was carried out by using probes for circ-ZEB1.33 and U6, the precipitation was amplified by using specific primer for 1: miR-200a-3p, 2: circ-ZEB1.33 (input), and 3: U6. **c** The prediction of potential miR-200a-3p binding to the 3′UTR of CDK6 was performed by using online tool TargetScan. **d** A luciferase reporter assay were performed for monitoring the CDK6 promoter activity regulated by WT and mutated 3′UTR of CDK6 in miR-200a-3p transfected Huh7 cells. The data were presented as mean ± SD, *P < 0.05, **P < 0.01, by Student’s t-test

To explore the mechanism of circ-ZEB1.33-miR-200a-3p-CDK6, we determined the expression of circ-ZEB-133 in various HCC cell lines (97H, Huh7, HepG2, SNU423, SNU475, and L02), we found that Huh7 and 97H were ranked first 2 high-expression cell lines of all seven (Fig. 3a). A RNA pull-down assay was performed by using a biotin-labeled probe of circ-ZEB1.33, a miR-200a-3p specific amplification fragment can be obtained after PCR the precipitated nuclear acid using miR-200a-3p specific primers (Fig. 3b). The possible binding site of miR-200a-3p to the 3′UTR of CDK6 was predicted by using Targetscan (http://www.targetscan.org/vert_71/), the binding site based CDK6 3′UTR mutation as well as a luciferase-based gene reporter assay were performed (Fig. 3c). The transfection of miR-200a-3p can significantly decrease the promoter activity but can be restored by using the 3′UTR mutation (Fig. 3d).

### Circ-ZEB1.33 promote HCC cell promotion by sponge of miR-200a-3p and up-regulating CDK6

**Fig. 4:**
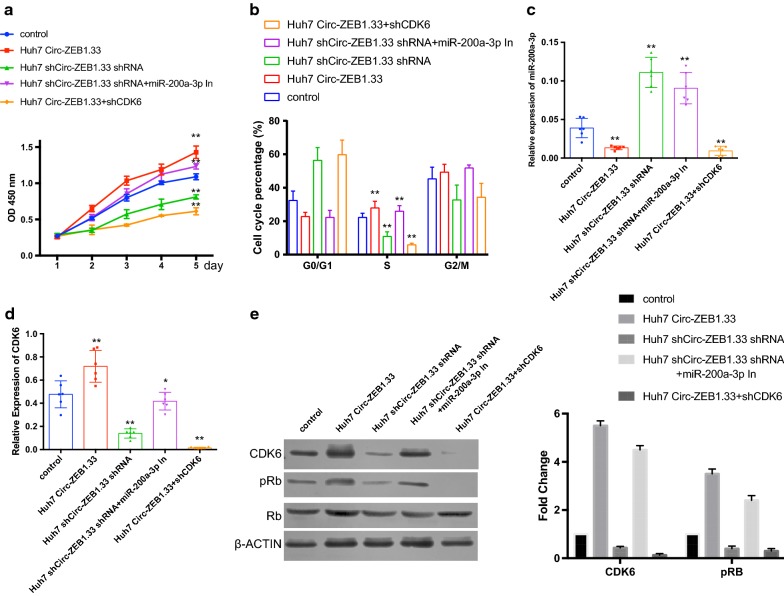
Circ-ZEB1.33 promote HCC cell promotion by sponge of miR-200a-3p and up-regulating CDK6. **a** The cell proliferation of Huh7 cells treated differently were monitored by using CCK8 assay. **b** The percentage of S phase in Huh7 cells treated differently was determined by using flow cytometry. **c**, **d** The transcription of miR-200a-3p and CDK6 in Huh7 cells treated differently were determined by using real-time PCR. **e** The protein expression of CDK6, p-Rb, and Rb in Huh7 cells treated differently by using western-blot. The data were presented as mean ± SD, *P < 0.05, **P < 0.01, by Student’s t-test

To investigate the effect of the circ-ZEB1.33-miR-200a-3p-CDK6 on the cell biology of HCC cell, we generated a serial of cell lines based on this regulatory axis including circ-ZEB1.33 overexpression (Huh7-circ-ZEB1.33), circ-ZEB1.33 knockdown (Huh7-shcirc-ZEB1.33), and circ-ZEB1.33 overexpression plus CDK6 knockdown (Huh7-circ-ZEB1.33+shCDK6). The proliferation was accessed by using CCK8 assay. On the one hand, overexpression circ-ZEB1.33 increased the cell proliferation significantly but can be decreased by knockdown CDK6 to significantly slower than the control group. On the other hand, the proliferation of Huh7-shcirc-ZEB1.33 was significantly decreased compared to the control group but can be restored by using the miR-200a-3p inhibitor (Fig. 4a). Also, the cell cycle of these cells was accessed, the percentage of S phase increased significantly in Huh7-circ-ZEB1.33 and decreased until significantly lower than the control group when CDK6 was knockdown in them. Also, the percentage of S phage decreased significantly in Huh7-shcirc-ZEB1.33 cells and further rescued by using an inhibitor to miR-200a-3p (Fig. 4b, Additional file 1: Figure S1). At last, the transcription of miR-200a-3p and CDK6, the protein expression of CDK6 were detected. The transcription of miR-200a-3p decreased with the overexpression of circ-ZEB1.33 and increased with circ-ZEB1.33 knockdown; there was no apparent effect on miR-200a-3p transcription when either its inhibitor or CDK6 was knockdown (Fig. 4c). Reversely to the transcription of miR-200a-3p transcription, CDK6 can be increased by overexpression of circ-ZEB1.33 while decreased by circ-ZEB1.33 knockdown, and this regulation was miR-200a-3p dependent (Fig. 4d and e). Moreover, the phosphorylated Rb in the residue of s795 also increased with the increased expression of CDK6 (Fig. 4e).

## Discussion

Our study found a circular RNA related regulatory axis: circ-ZEB1.33-miR-200a-3p-CDK6 in human HCC. Excessive expression of circ-ZEB1.33 was related to the increased proliferation of HCC cells due to increased expression of CDK6. Also, the circ-ZEB1.33 expression was seriously related to the HCC progression, because its expression was significantly higher in TMN III–IV compared to TMN I–II. We noticed that cir-ZEB1.33 also overexpressed in the serum of HCC patients, and was related to the progress and prognosis of HCC. Therefore, the expression of circ-ZEB1.33 or even the circ-ZEB1.33-miR-200a-3p-CDK6 axis can serve as a potential target for HCC diagnosis. CDK6 is a classic cell cycle-related protein, regulated by cyclins, more specifically by Cyclin D and Cyclin-dependent kinase inhibitor proteins [5, 6]. It is a member of the cyclin-dependent kinase (CDK) family which are known to be important regulators of cell cycle progression [7]. CDK6 was one of the components of a kinase complex which is important for G1 phase progression and G1/S transition. The complex is also composed by an activating sub-unit, the cyclin D. This kinase has been shown to phosphorylate tumor suppressor protein, Rb, making CDK6 an important protein in cancer development [8]. In our in vitro study, we found that increased CDK6 expression in a Huh7 cell can enhance the phosphorylation of Rb in the residue of S795, thus to release E2F, an S phase-related transcriptional factor, promoting cell cycle into S phase [9].

CDK6 and other regulators of the G1 phase of the cell cycle are known to be unbalanced in more than 80–90% of tumors [10, 11]. Also, many research indicated that CDK6 was overexpressed and can act as a potential biomarker for human HCC [12, 13]. However, the expression of CDK6 in the tumor can’t access without the tumor tissues. In the present study, we also found that the overexpression of CDK6 in human HCC tissues was due and correlated to the overexpression of a circulating RNA, circ-ZEB1.33, not only in the tumor, but also in the serum. The detection of serum circ-ZEB1.33 can reflect the CDK6 level in the tumor tissue, so we think serum circ-ZEB1.33 is a potentially valuable biomarker for HCC diagnosis. Furthermore, the serum circ-ZEB1.33 can serve as an indicator for prognosis of the post-surgery HCC patients. However, we found more robust proliferation decreasing in the CDK6 shRNA cells compared to the circ-ZEB1.33 shRNA cells, this result might indicate that there might be other mechanism besides the circ-ZEB1.33/miR-200-3p in the regulation of CDK6.

Besides its diagnosis value, CDK6 is also one of the pharmacological targets designated for molecular cancer therapy [14, 15]. Unlike the thoughts of direct inhibition of CDK6 activity, present results might provide another clue. We found that the HCC CDK6 can be regulated by suppressing the expression of circ-ZEB1.33. Also, the proliferation ability can be effectively inhibited by knockdown the expression of circ-ZEB1.33 and the regulation is miR-200a-3p dependent.

HCC CDK6 can be decreased by miR-200a-3p through binding to its 3′UTR. Similar results have been proved in melanoma, Matias et al. found that significant low microRNA-200a expression in metastatic melanoma cells, this downregulation of miR-200a results in higher levels of CDK6 and a more significant response to CDK4/6 inhibitors [16]. Within the HCC tissues, we also found that miR-200a-3p decreased, which is because circ-ZEB1.33 can sponge miR-200a-3p by their complementary sequence. The conclusion can be proved by the RNA pull-down assay and the circ-ZEB 1.33 Knockdown assay.

## Conclusion

In summary, circ-ZEB1.33 is a tumor promotion circular RNA, promoting HCC cell proliferation by enhancing the expression of CDK6 through sponging miR-200a-3p. The secreted circ-ZEB1.33 can be detected in the human serum, serving as a valuable biomarker in HCC diagnosis and prognosis prediction.

## Additional file

**Additional file 1: Figure S1.** Flow cytometry analysis of the Huh7 cell cycle treated differently indicated in the figure.
