# Supplementary material for: Circ-ZEB1.33 promotes the proliferation of human HCC by sponging miR-200a-3p and upregulating CDK6
Source: Cancer Cell Int. 2018 Aug 13;18:116. doi: 10.1186/s12935-018-0602-3 (PMC6090603; doi:10.1186/s12935-018-0602-3)
Supplement: Supplementary file 1 — Additional file 1: Figure S1. Flow cytometry analysis of the Huh7 cell cycle treated differently indicated in the figure. [file 12935_2018_602_MOESM1_ESM.docx]

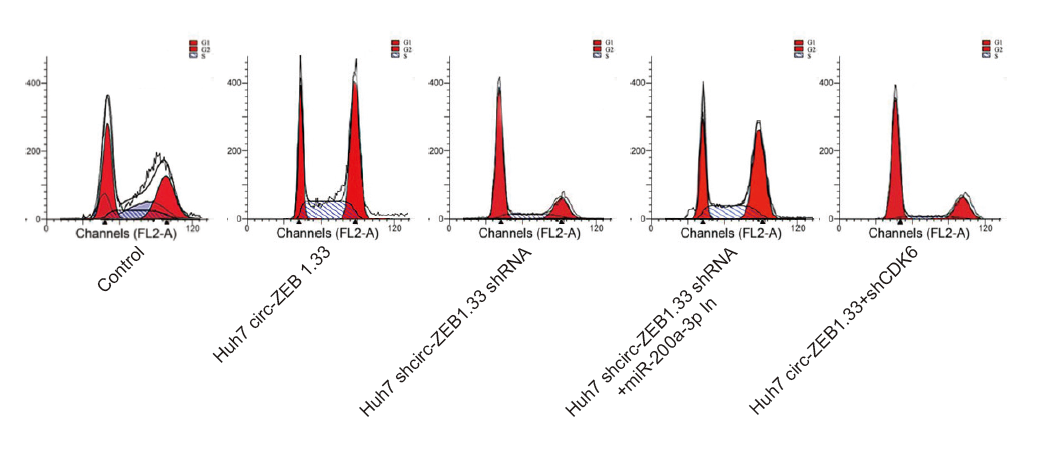


Figure S1

Flow cytometry analysis of the Huh7 cell cycle treated differently indicated in the figure.
